# The Reproducibility of 4-km Time Trial (TT) Performance Following Individualised Sodium Bicarbonate Supplementation: a Randomised Controlled Trial in Trained Cyclists

**DOI:** 10.1186/s40798-017-0101-4

**Published:** 2017-09-21

**Authors:** Lewis Anthony Gough, Sanjoy Kumar Deb, Andy Sparks, Lars Robert McNaughton

**Affiliations:** Sports Nutrition and Performance Group, Department of Sport and Physical Activity, Edge Hill University, Ormskirk, Lancashire L39 4QP UK

**Keywords:** Buffering, Alkalosis, Performance, Acid-base balance, Ergogenic aids, Reliability

## Abstract

**Background:**

Individual time to peak blood bicarbonate (HCO_3_^−^) has demonstrated good to excellent reproducibility following ingestion of both 0.2 g kg^−1^ body mass (BM) and 0.3 g kg^−1^ BM sodium bicarbonate (NaHCO_3_), but the consistency of the time trial (TT) performance response using such an individualised NaHCO_3_ ingestion strategy remains unknown. This study therefore evaluated the reproducibility of 4-km TT performance following NaHCO_3_ ingestion individualised to time to peak blood bicarbonate.

**Methods:**

Eleven trained male cyclists completed five randomised treatments with prior ingestion of 0.2 g kg^−1^ (SBC2) or 0.3 g kg^−1^ BM (SBC3) NaHCO_3_, on two separate occasions each, or a control trial entailing no supplementation. Participants completed a 4-km cycling TT on a Velotron ergometer where time to complete, power and speed were measured, whilst acid-base blood parameters were also recorded (pH and blood bicarbonate concentration HCO_3_^−^) and lactate [La^−^].

**Results:**

Alkalosis was achieved prior to exercise in both SBC2 and SBC3, as pH and HCO_3_^−^ were greater compared to baseline (*p* < 0.001), with no differences between treatments (*p* > 0.05). The reproducibility of the mean absolute change from baseline to peak in HCO_3_^−^ was good in SBC2 (*r* = 0.68) and excellent in SBC3 (*r* = 0.78). The performance responses following both SBC2 and SBC3 displayed excellent reproducibility (*r* range = 0.97 to 0.99).

**Conclusions:**

Results demonstrate excellent reproducibility of exercise performance following individualised NaHCO_3_ ingestion, which is due to the high reproducibility of blood acid-base variables with repeat administration of NaHCO_3_. Using a time to peak HCO_3_^-^ strategy seems to cause no dose-dependent effects on performance for exercise of this duration and intensity; therefore, athletes may consider smaller doses of NaHCO_3_ to mitigate gastrointestinal (GI) discomfort.

## Key Points

- Time to peak blood bicarbonate (HCO_3_^−^) induced via ingestion of sodium bicarbonate (NaHCO_3_) produces consistent physiological responses prior to, during and post-exercise.
- Four-kilometre TT performance displays excellent reliability following repeat administration of 0.2 and 0.3 g kg body mass (BM) NaHCO_3_ individualised to time to peak HCO_3_^−^.
- Athletes need to be aware that the gastrointestinal (GI) discomfort response is varied even with repeated administration; therefore, this needs to be monitored.

## Background

Personalised nutrition has gathered recent attention as individual characteristics (i.e. genetics, training status and nutrition) can potentially improve or reduce the physiological adaptation from the same intervention [21]. Through personalising nutrition strategies, both the individual bioavailability and bioefficacy can be quantified to maximise the effects of an intervention within individuals [23]. This concept can also be applied to the use of nutritional ergogenic aids aimed to enhance human physiology and exercise performance; however, common practice of ergogenic aid research determines the effectiveness of a single treatment by evaluating the mean differences between groups or trials [15]. As a result, the inter- and intra-individual responses are seldom considered. To account for this, repeat administration of the experimental treatment is plausible, thereby allowing assessment of the reproducibility of nutritional ergogenic aids [18, 19]. This method permits athletes to appropriately evaluate the effectiveness of ergogenic aids and to assess if they can provide consistent benefits to exercise performance upon repeated use during training and/or competition [4].

The use of over-the-counter sodium bicarbonate (NaHCO_3_) is one example of a supplement that allows for repeated administration by representing a low cost and easy digestion. Primarily, NaHCO_3_ is used as an ergogenic aid to mitigate the effects of metabolic acidosis (i.e. decline in pH) by increasing bicarbonate (HCO_3_^−^) buffering capacity during short distance/duration high-intensity exercise of between 1 and 10 min [27]. Traditionally, NaHCO_3_ is administered in a single 0.3 g kg^−1^ body mass (BM) dose at a standardised time prior to exercise ranging between 1 and 4 h [26, 32, 34]. It was originally suggested that the point of peak alkalosis (measured by either peak pH and/or HCO_3_^−^) occurred between 60 and 90 min post NaHCO_3_ ingestion [29, 32, 34]. This same peak has also been suggested to occur up to 180 min in other studies however [6, 22, 35], whilst more recently, it has been shown to range between 40 and 140 min following both 0.2 and 0.3 g kg^−1^ BM NaHCO_3_ [14]. These data therefore suggest that there is a large individual variation in time to peak alkalosis. Consequently, some athletes may not be ingesting this supplement at a time that corresponds to their peak buffering capacity, which might limit the resulting ergogenic effect.

To account for such inter-individual variation an individualised NaHCO_3_ strategy has recently been recommended, which entails administering NaHCO_3_ at a time point to ensure peak pH and/or HCO_3_^−^ concentrations at the beginning of exercise [10, 14, 22, 28, 33]. By identifying peak alkalosis, it ensures that the HCO_3_^−^ buffering capacity is maximised, compared to a standardised ingestion strategy where some participants may not begin exercise at peak alkalosis. This methodological alteration may therefore account for some of the equivocal performance data which has been observed using NaHCO_3_ [5, 13, 16, 32]. Gough et al. [14] have displayed using an individualised NaHCO_3_ time to peak strategy that time to peak blood HCO_3_^-^ was highly repeatable using intraclass correlation coefficient (ICC) analysis (*r* = 0.77 to 0.94). The authors also reported the mean HCO_3_^−^ change from baseline to peak was + 5.7 mmol l^−1^ following 0.2 g kg^−1^ BM NaHCO_3_, which is greater than the + 3.9 ± 0.9 mmol l^−1^ mean change reported in a meta-analysis from a 0.3 g kg^−1^ BM dose [5]. This suggests that using an individualised NaHCO_3_ ingestion strategy may elicit more reproducible biological acid-base balance changes, whilst a smaller 0.2 g kg^−1^ BM NaHCO_3_ dose induces a change in HCO_3_^−^ greater than the mean change observed using a standardised 0.3 g kg^−1^ BM NaHCO_3_ dose. This smaller dose therefore represents a viable option for the athlete, and the performance responses following this dose are worthwhile to determine.

A factor that may hamper the use of NaHCO_3_ in a practical sense is the onset of gastrointestinal (GI) discomfort. Common symptoms include nausea, belching, diarrhoea and vomiting, which can have both practical and perceived impacts on the efficacy of this supplement for performance enhancements [32]. Therefore, whilst it is important to initially heighten the level of peak alkalosis, it is of equal importance to mitigate GI discomfort. One strategy to reduce the severity of GI discomfort is a lower dose (0.2 g^.^kg^-1^ BM), which has been shown to reduce the severity of the symptom suffered compared to 0.3 g kg^−1^ BM [13, 26]. This dose might not have been used as widely in previous research due to the previous standardised times of ingestion, and the early findings of McNaughton [26] reporting 0.3 g kg^−1^ BM produced greater ergogenic benefits compared to 0.2 g kg^−1^ BM NaHCO_3_. Nonetheless, with an individualised ingestion strategy, an increase in HCO_3_^−^ (~ 5 mmol l^−1^) should be reached with a lower amount of NaHCO_3_ (i.e. 0.2 g kg^−1^ BM NaHCO_3_), whilst also reducing the severity of GI discomfort [14, 22].

In studies utilising standardised NaHCO_3_ ingestion strategies, equivocal performance responses have been reported across consecutive repeated trials [6, 12]. Dias et al. [12] reported when four seperate cycling bouts at 110% peak power output until time to exhaustion (TTE) were performed with ingestion of 0.3 g kg^−1^ BM NaHCO_3_, ten participants improved performance in at least one trial, whilst only one participant improved in all trials. Although blood pH and HCO_3_^−^ were generally repeatable in this study, a factor contributing to this variation may have been the exercise test employed. A high variation in responses has been displayed in TTE tests [17], particularly in untrained individuals which feature in the Dias et al. [12] study, and compared to time trial (TT) tests [7, 9, 16, 24]. The lack of consistent improvements in response to NaHCO_3_ may therefore be due to the exercise protocol adopted, not the failure of NaHCO_3_’s buffering mechanism to enhance performance. In a further study, Carr et al. [6] reported that performance responses were repeatable for mean power during a 2000-m rowing TT performance displaying a typical error (TE) of 2.1%. The authors, however, reported the change in HCO_3_^−^ from baseline to pre-exercise displayed a very large TE (2.5%) and coefficient of variation (CV) (7%), which may explain why no performance effect was observed. This evidence suggests the blood responses following NaHCO_3_ may not be consistent following 0.3 g kg^−1^ BM NaHCO_3_. Furthermore, both Dias et al. [12] and Carr et al. [6] utilised a standardised NaHCO_3_ ingestion strategy and only reported mean blood responses, which may have adversely affected the blood and performance responses and thus, the interpretation of the data. Only two studies have evaluated the repeatability of the performance responses following acute ingestion of NaHCO_3_ and reported equivocal findings, meaning further enquiry is required. The aim of this study was therefore to investigate the reproducibility of blood acid-base, performance and GI discomfort responses following two individualised NaHCO_3_ doses.

## Methods

### Participants

Eleven trained male cyclists volunteered for a randomised, double-blind, crossover design study (height 182 ± 8 cm, body mass 86.4 ± 12.9 kg, age 32 ± 9 years, maximal oxygen consumption (VO_2max_) 58.0 ± 3.8 ml kg min^−1^, peak power 4.5 ± 0.5 W kg^−1^). The participant inclusion required individuals to meet the criteria of ‘trained cyclist’ as outlined by De Pauw et al. [11], be between 18 and 50 years of age, training for a minimum of 4 h week^−1^ and have 2 years continuous cycling experience. Participants were also excluded if they had ingested any intra/extracellular buffer in the prior 6 months before data collection. Ethical approval was obtained from the Departmental Research Ethics Committee, and each participant provided written informed consent prior to any data collection, with the research conducted in accordance with the Declaration of Helsinki.

Participants visited the laboratory in a 4-h postprandial state to limit confounding nutritional effects on exercise performance and at the same time of day to control for circadian rhythms [30]. Participation in any strenuous/unaccustomed exercise and alcohol intake was prohibited for the 24 h prior to each treatment arm. Caffeine was also to be avoided for the 12 h prior. Compliance with the above procedures was checked via written logs of nutritional intake, and participants were asked to replicate nutritional practices for subsequent trials (adherence = 100%).

### Determination of Maximal Oxygen Consumption and Time to Peak Blood Bicarbonate

Participants initially completed an incremental exercise test on an electromagnetically braked cycle ergometer (Excalibur Sport, Lode, Netherlands) to determine VO_2max_ and peak power output (PPO). After a self-selected warm-up, the cycling protocol began at 75 W for 1 min and then increased by 1 W every 2 s (30 W min^−1^) until the point of volitional exhaustion. This was determined by the inability for the participant to sustain their self-selected cadence for longer than 5 s, whereby they were given an initial warning, and the test was then terminated on the subsequent occurrence.

Two trials were conducted in a randomised order to identify time to peak blood HCO_3_^−^ following ingestion of either 0.2 g kg^−1^ NaHCO_3_ (SBC2) or 0.3 g kg^−1^ BM NaHCO_3_ (SBC3). This was ingested as a drink and mixed with 400 ml of water and 50 ml double strength blackcurrant sugar-free squash. The use of time to peak HCO_3_^−^ was utilised as this has displayed greater reproducibility compared to time to peak pH [14]. Finger prick capillary blood samples were taken prior to NaHCO_3_ ingestion in a seated position and collected within a 100-μl heparin-coated glass clinitube for analysis of blood pH and HCO_3_^−^ (ABL800 BASIC, Radiometer Medical Ltd., Denmark). Subsequent blood samples were drawn every 10 min over a 180-min period to identify time to peak HCO_3_^−^. The double-blind nature of the study was maintained by a volunteer outside of the research team, who identified the time to peak HCO_3_^−^.

### Familiarisation and Experimental Treatment Arms

The participants then performed a 4-km TT familiarisation trial, followed by a further five separate visits requiring completion of the same exercise following ingestion of either 0.2 g kg^−1^ BM NaHCO_3_ twice (SBC2a and SBC2b), 0.3 g kg^−1^ BM NaHCO_3_ twice (SBC3a and SBC3b) or a control treatment entailing no supplementation in a randomised order. A control trial was conducted to obtain a reference point measure of performance. Blood samples were taken at rest, at time to peak HCO_3_^−^ and post-exercise to analyse blood pH and HCO_3_^−^, with the addition of a 5-μl sample for blood lactate [La^−^] (Lactate Pro 2, Arkray, Japan). Ingestion of either SBC2 or SBC3 was completed within 10 min, and participants remained seated and rested until their respective time to peak. A questionnaire to assess the severity gastrointestinal (GI) discomfort was used every 10 min until individual time to peak HCO_3_^−^ [14, 28].

Each TT was completed on a Velotron Racermate™ cycle ergometer interfaced with Velotron 3D coaching software (Racermate, USA). Frame geometry (handlebars, seat position) and gear ratios were selected by the participant to match their preferred riding style to be replicated for each subsequent trial. Strong and consistent verbal encouragement was provided throughout the TT. Participants were blinded from the clock, but were provided with feedback on distance covered, watts (W) and cadence (rev min^−1^) via the display [38]. This feedback was provided as cyclists used in this study were not habitually completing 4-km TT distances in competition.

### Statistical Analysis

Shapiro-Wilk test and standard graphical methods (Q-Q plots) were used to assess normality of the data, and the Mauchly test was used for homogeneity and variance. Both the severity and time to peak GI discomfort were considered non-normally distributed by the respective normality tests. Therefore, a Friedman rank test was used as an alternative and is reported with *z* score (significance) and effect size (*d*) calculated by *Z*/√*n*. Interpretation was then considered as small (0.10), medium (0.24) and large (> 0.37) [20]. Mean speed, power and time to complete were compared between treatment arms using a repeated measures analysis of variance (ANOVA). Haematological data such as pH, HCO_3_^−^ and [La^−^] were analysed using a two-way (treatment × time) repeated measures ANOVA. Post hoc comparisons were determined by Bonferroni correction. Effect size is reported as partial *η* ^2^ value and considered trivial (< 0.20), small (0.20–0.49), moderate (0.50–0.79) and large (≥ 0.80) in accordance with the conventional Cohen’s *d* interpretations [8].

Limits of agreement (LOA) with 95% percent limits and Bland-Altman plots were initially used to determine if data was heteroscedastic [3]. The repeatability of both blood acid-base balance and performance responses to SBC2 and SBC3 was determined using intraclass correlation coefficients (ICC) with *r* value and significance level (i.e. *p* value) as per previous recommendations [2, 39]. Interpretation of reproducibility was categorised by the respective *r* value with categories of poor (*r* ≤ 0.40), fair (*r* = 0.40–0.59), good (*r* = 0.60–0.74) and excellent (*r* ≥ 0.74). Typical error (TE) is reported and calculated using the method of Hopkins [19], with categories of small (≤ 0.2–0.6), moderate (0.6–1.2), large (1.2–2.0), very large (2.0–4.0) and extremely large (≥ 4.0%) used to interpret the data. Coefficient of variation (CV) was reported using SD/mean × 100. Statistical procedures were completed using SPSS version 22 (IBM, Chicago, USA), and calculations were carried out using Microsoft Excel 2013 (Microsoft Inc., USA). All data are presented as mean ± SD unless otherwise stated. Statistical significance was set at *p* > 0.05.

## Results

### Reliability of Treatments

The blood responses following SBC treatments were largely reproducible (Tables 1 and 2). Individual blood responses displaying absolute changes from baseline to peak pH and HCO_3_^−^ are depicted in Table 3. The only inconsistency observed was the absolute change in pH from baseline to peak in SBC3, revealing a poor ICC. Excluding that case, blood responses pre-, during and post-exercise ranged from good to excellent for all SBC treatments (ICC range *r* = 0.68 to 0.95).

**Table 1:** Overview of intraclass correlation coefficients (ICC) and typical error (TE) statistical analysis following sodium bicarbonate (NaHCO_3_) treatments (SBC2 and SBC3) on pre-, during and post-exercise blood pH, bicarbonate (HCO_3_^−^) and lactate

Absolute change from baseline to peak (including initial TTP trial)								
HCO_3_^−^	SBC2	SBC3	pH	SBC2	SBC3			
ICC			ICC					
*r* value	0.79	0.69	*r* value	0.79	0.17			
*p* value	< 0.001	0.006	*p* value	< 0.001	0.326			
Typical error %	1.2	1.4	Typical error %	1.4	1.7			
Interpretation	Excellent	Good	Interpretation	Excellent	Poor			
During exercise								
Decline in HCO_3_^−^	SBC2	SBC3						
ICC								
*r* value	0.69	0.95						
*p* value	0.026	< 0.001						
Typical error %	1.3	0.6						
Interpretation	Good	Excellent						
Post-exercise								
Blood lactate	SBC2	SBC3	HCO_3_^−^	SBC2	SBC3	pH	SBC2	SBC3
ICC			ICC			ICC		
*r* value	0.91	0.92	*r* value	0.89	0.95	*r* value	0.92	0.95
*p* value	< 0.001	< 0.001	*p* value	< 0.001	< 0.001	*p* value	< 0.001	< 0.001
Typical error %	0.7	0.8	Typical error %	0.8	0.6	Typical error %	0.6	0.6
Interpretation	Excellent	Excellent	Interpretation	Excellent	Excellent	Interpretation	Excellent	Excellent

**Table 2:** Mean (± SD) blood pH, bicarbonate (HCO_3_^−^) and lactate responses following sodium bicarbonate (NaHCO_3_)

Baseline to pre-exercise	Ind. time to peak SBC2	SBC2	SBC2	Ind. time to peak SBC3	SBC3	SBC3
pH	0.07 ± 0.02	0.06 ± 0.01	0.07 ± 0.02	0.09 ± 0.03	0.06 ± 0.02	0.07 ± 0.02
HCO_3_^−^ (mmol l^−1^)	5.5 ± 0.7	4.9 ± 0.8	5.2 ± 1.1	6.5 ± 1.3	5.6 ± 1.0	5.7 ± 1.1
During exercise						
HCO_3_^−^ usage (mmol l^−1^)		12.7 ± 2.6	13.9 ± 1.7		13.8 ± 2.7	13.2 ± 2.5
Post-exercise						
Blood lactate (mmol l^−1^)		16.1 ± 3.4	17.2 ± 3.7		16.1 ± 3.4	16.3 ± 3.8

**Table 3:** Individual blood responses for both blood pH and bicarbonate (HCO_3_^−^) following sodium bicarbonate (NaHCO_3_)

Blood HCO_3_^−^	Absolute change in mmol l^−1^							Blood pH	Absolute change					
Participant number	Ind. dose	SBC2	SBC2	Ind. dose	SBC3	SBC3	CON	Ind. dose	SBC2	SBC2	Ind. dose	SBC3	SBC3	CON
1	5.5	5.4	5.7	4.9	4.1	4.8	0.0	0.06	0.07	0.04	0.09	0.09	0.08	0.00
2	6.8	5.6	5.1	5.6	4.1	4.9	0.1	0.08	0.08	0.07	0.05	0.04	0.05	0.00
3	5.6	4.7	7.2	5.0	6.2	6.9	− 0.5	0.07	0.05	0.06	0.07	0.05	0.08	0.00
4	6.2	4.5	5.0	8.1	7.2	7.2	0.0	0.05	0.03	0.08	0.10	0.02	0.03	0.01
5	5.8	5.5	6.3	8.1	5.8	4.9	0.0	0.09	0.05	0.06	0.06	0.08	0.07	0.00
6	5.3	5.4	5.0	6.5	5.4	6.9	0.0	0.09	0.08	0.11	0.16	0.05	0.04	0.00
7	4.7	3.3	2.9	7.6	6.5	4.2	0.1	0.07	0.06	0.06	0.10	0.07	0.08	0.00
8	3.8	3.8	4.3	7.8	6.4	6.1	− 0.1	0.06	0.05	0.05	0.09	0.06	0.06	0.00
9	5.3	5.6	5.2	4.6	4.8	4.8	0.3	0.05	0.06	0.06	0.06	0.09	0.09	0.00
10	5.4	5.8	6.0	7.0	6.0	6.9	0.0	0.05	0.04	0.04	0.09	0.05	0.04	0.00
11	5.9	4.9	5.2	6.4	5.6	5.7	0.0	0.11	0.07	0.12	0.09	0.10	0.11	0.01
Mean	5.5	5.0	5.3	6.5	5.6	5.8	0.0	0.07	0.06	0.07	0.09	0.06	0.07	0.00
SD	0.7	0.8	1.1	1.3	0.9	1.0	0.2	0.02	0.02	0.02	0.03	0.02	0.02	0.00

Excellent reproducibility of time to complete the 4-km TT was observed in both SBC2 (*r* = 0.97, *p* < 0.001) and SBC3 (*r* = 0.99, *p* < 0.001) with a low TE (range = 0.3 to 0.5%; Table 4). Mean power displayed excellent reproducibility in both SBC2 (*r* = 0.96, *p* < 0.001; TE = 0.6%) and SBC3 (*r* = 0.98, *p* < 0.001; TE = 0.4%). Mean speed also displayed excellent reproducibility in SBC2 (*r =* 0.97, *p* < 0.001; TE = 0.6%) and SBC3 (*r* = 0.98, *p* < 0.001; TE = 0.4%). Eight participants reported symptoms of GI discomfort (Table 5). The severity of GI discomfort displayed good reproducibility in SBC2 (*r =* 0.72, *p* = 0.023; TE = 1.3%) in comparison to excellent in SBC3 (*r* = 0.76, *p* = 0.017; TE = 1.3%). The time to peak GI discomfort displayed excellent reproducibility in both SBC2 (*r =* 0.99, *p* < 0.001; TE = 0.2%) and SBC3 (*r =* 0.84, *p* = 0.005; TE = 1.1%).

**Table 4:** Individual 4-km TT performance differences between sodium bicarbonate (NaHCO_3_) treatments (SBC2 and SBC3)

Participant number	Difference in seconds between treatments		% difference between treatments	
	SBC2 vs. SBC2	SBC3 vs. SBC3	SBC2 vs. SBC2	SBC3 vs. SBC3
1	1.4	2.3	0.4	0.6
2	2.5	1.1	0.7	0.3
3	0.5	3.0	0.1	0.9
4	11.2	4.3	2.9	1.1
5	0.2	0.4	0.1	0.1
6	4.6	0.4	1.2	0.1
7	5.6	1.9	1.5	0.5
8	7.8	2.5	2.1	0.7
9	3.0	5.2	0.8	1.3
10	1.6	2.4	0.4	0.7
11	2.7	1.7	0.7	0.4
Mean	3.7	2.3	1.0	0.6
SD	3.2	1.4	0.8	0.4
TE %	0.5		0.3

**Table 5:** Individual severity and time to peak gastrointestinal (GI) responses in participants who reported symptoms only (*n* = 8)

	SBC2			SBC2			SBC3			SBC3		
Participant number	Severity	Peak GI (min)	Symptom	Severity	Peak GI (min)	Symptom	Severity	Peak GI (min)	Symptom	Severity	Peak GI (min)	Symptom
1	6	30	Diarrhoea/bowel urgency	2	30	Belching	4	50	Belching	3	60	Belching
2	3	30	Belching	3.5	20	Belching	0	0	None	0	0	None
3	10	50	Diarrhoea/bowel urgency	3	40	Belching	5	60	Belching	0	0	None
4	3	70	Stomach bloating	3	70	Stomach bloating	10	60	Diarrhoea/bowel urgency	10	90	Diarrhoea/bowel urgency
5	0	0	None	0	0	None	3	80	Stomach cramp	10	80	Diarrhoea/bowel urgency
6	0	0	None	0	0	None	3	40	Nausea/bloating	4	40	Diarrhoea/bowel urgency
7	0	0	None	0	0	None	2	40	Bloating	5.5	30	Bloating/belching
8	0	0	None	0	0	None	0	0	None	4	40	Stomach cramp
Mean	2.8	23		1.4	20		3.4	41		4.6	43	
SD	3.4	25		1.5	24		3.0	27		3.6	31	

### Effect of Treatment

In the initial treatments to identify time to peak alkalosis, peak HCO_3_^−^ was achieved between 40 and 110 min (median 50 min) following SBC2 and between 40 and 100 min (median 70 min) following SBC3. There was a large inter-individual variation as the CV range was between 27 and 33% for SBC treatments. A + 5.5 ± 0.7 mmol l^−1^ change in HCO_3_^−^ from baseline to peak was observed following SBC2, compared to + 6.5 ± 1.3 mmol l^−1^ in SBC3, respectively (Table 2). Both SBC treatments displayed a large CV ranging from 14 to 19%. In comparison, peak pH from baseline was achieved between 50 and 80 min (median 60 min) following SBC2 and between 50 and 90 min (median 70 min) following SBC3. Similarly, a large CV range was also observed (18 to 22%). The change from baseline to peak was + 0.07 ± 0.02 in SBC2 and + 0.09 ± 0.03 in SBC3, again with a large CV from 29 to 34% (Table 2).

Following NaHCO_3_ ingestion, HCO_3_^−^ was greater compared to baseline in both SBC2 and SBC3, revealing a main effect for time (*p* < 0.001, *η* ^2^ = 0.932); however, no difference between treatments was observed (*p* = 0.184, *η* ^2^ = 0.010; Table 2). This trend was similar for pH with a main effect for time (*p* ≤ 0.001, *η* ^2^ = 0.983) and no difference between SBC treatments (*p* = 0.512, *η* ^2^ = 0.003). During exercise, there was no difference in the decline of HCO_3_^−^ following both SBC2 and SBC3 (*p* = 0.251, *η* ^2^ = 0.129). Post-exercise [La^−^] also displayed no difference between SBC treatments (*p* = 0.494, *η* ^2^ = 0.076; Table 2).

The time to complete the 4 km distance was similar for both SBC2 (combined mean = 373.6 ± 13.3 s) and SBC3 (373.5 ± 13.1 s) (mean diff = 0.01 s; *p* = 0.929, *η* ^2^ = 0.015; Table 3). Compared to the control treatment arm, both SBC2 and SBC3 produced faster completion times (mean difference SBC2 = − 8.0, *p* ≤ 0.001 and 8.8 s, *p* = 0.006; SBC3 = − 8.2, *p* = 0.005 and 8.6 s, *p* = 0.006). There was no difference between SBC2 and SBC3 for either mean power (*p* = 0.966, *η* ^2^ = 0.009) or mean speed (*p* = 0.746, *η* ^2^ = 0.040). Severity of GI discomfort in SBC3 was marginally greater (3.4 ± 3.0 and 4.6 ± 3.6) compared to SBC2 (2.8 ± 3.4 and 1.4 ± 1.5), however not significantly (*z* = 0.268, *d* = 0.08) (Table 5). Likewise, time to peak GI discomfort was around 20 min later in SBC3 (41 ± 27 and 43 ± 31 min) compared to SBC2 (23 ± 25 and 20 ± 24 min), although again not significantly (*z* = 0.197, *d* = 0.06; Table 5).

## Discussion

The aim of this study was to investigate the reproducibility of both blood acid-base analytes and performance responses following repeated ingestion of NaHCO_3_. The present study is the first to demonstrate that both the physiological and performance responses are reproducible when the ingestion time for NaHCO_3_ is determined by individual time to peak HCO_3_^−^. This provides a legitimate and workable strategy to elicit consistent performance responses on exercise of this duration and intensity. The primary findings are in contrast to those of the previous research which has reported large inter- and intra-individual variability in both performance responses [12] and blood acid-base responses [6] following a standardised NaHCO_3_ ingestion strategy. It would therefore appear that an individual time to peak HCO_3_^−^ ingestion strategy is more efficacious in eliciting consistent responses.

Performance following NaHCO_3_ displayed excellent reproducibility within the trained cyclist cohort used in this study. The TE values for the time to complete the 4-km cycling TT in the present study are consistent with a previous investigation of the reliability of TT cycling of the same distance without NaHCO_3_ ingestion. Stone et al. [36] reported a TE of 0.9% which is similar to both the SBC conditions in the present study (TE ≤ 0.5%), whilst mean speed also displayed similar TEs (< 1%). These consistent values may be evident due to the high training status of the cyclists used in each respective study and the well-reported reliability of 4 km TT cycling performance under laboratory conditions [1, 23, 38]. The present study data suggests the inclusion of NaHCO_3_ did not compromise the reliability of the performance responses, and therefore, it can be recommended to elicit consistent performance responses during this type of exercise. The present study also displays the intra-individual variation between conditions (i.e. SBC2 vs. SBC2). The intra-individual variation was larger in SBC2 than in SBC3, although this was largely due to participant 4 who displayed an 11-s difference between the two SBC2 treatments. With participant 4 removed from the analysis, a 3-s change would have occurred which is more akin to SBC3. In contrast, four of the sample reported a difference < 1 s between SBC conditions. Athletes should therefore be aware of this variation and monitor accordingly if they consider using NaHCO_3_ on a consistent basis.

The primary findings agree with Carr et al. [6] who reported a low TE (2.1%) for 2000 m rowing ergometry following NaHCO_3_. In contrast, a lack of repeatability was observed in a later study [12] reporting a mean CV of 7.4 ± 3.2% (range = 2.5 to 14.8%) and large intra-individual variation following NaHCO_3_. This is considerably higher than the CV of Carr et al. [6] (1.6%) and the present study (3.5%), which is likely due to the lower training status of the participants used by Dias et al. [12]. Equally, both the study of Carr et al. [6] and the present study used TT simulation compared to a cycling TTE protocol at 110% peak power output [12]. It is suggested the ‘open-ended’ nature, the lack of control over power output and motivational differences during a TTE test explain the greater variation compared to TT simulation [24]. In a study by Saunders et al. [31], however, an identical protocol as used by Dias et al. [12] displayed excellent test-retest reliability in ICC analysis (*r* = 0.88), suggesting the exercise protocol did not negate the reliability of responses following NaHCO_3_. It is more likely, therefore, that the training status of participants may have caused the greater variation in responses compared to that of Carr et al.’s [6] and the present study, as a trained athlete is likely to be able to produce a similar effort compared to a recreationally active participant.

The present study reports similar reproducibility of blood acid-base balance variables following NaHCO_3_ in previous studies [6, 12]. Carr et al. [6] reported a high variation in HCO_3_^−^ post-supplementation (TE = 2.4%), whilst Dias et al. [12] highlighted a discrepancy of 0.02 pH units between two NaHCO_3_ treatment arms. Likewise, the present study reported similar inconsistencies such as a 0.03 pH unit discrepancy between the SBC3 individual TTP experiment and SBC3, and a 0.9 mmol l^−1^ HCO_3_^−^ discrepancy in the same SBC3 treatment. Whether such small discrepencies are practically meaningful however, is abtruse. Nontetheless, individual analysis revealed a small number of inconsistencies in blood acid-base balance, as participant 4’s absolute HCO_3_^−^ change from baseline to peak was + 4.7 mmol l^−1^ in SBC2a, compared to a + 7.2 mmol l^−1^ change in SBC2b. Likewise, the change from baseline to peak for participant 8 was + 2.3 mmol l^−1^ different in SBC3 (+ 6.5 vs. + 4.2 mmol l^−1^). Some degree of inconsistency was subsequently evident in performance times, particularly with participant 4, who was around 11 s slower in SBC2a than in SBC2b, whereas participant 8 was slower in SBC3a by around 3 s, despite the greater change in HCO_3_^−^, compared to SBC3b. These inconsistencies add to previous findings that have reported whilst the majority of blood responses were reproducible, some individuals display large variability following NaHCO_3_ [14]. Given these inconsistencies, individuals should monitor the performance effects following NaHCO_3_ ingestion across multiple trials to ensure similar responses are elicited.

The physiological responses pre, during and post the TTs displayed good to excellent reproducibility following both NaHCO_3_ doses used in this study. During exercise, the change in HCO_3_^−^ was highly repeatable for all SBC conditions and the values were reflective of a recent study following NaHCO_3_ during a 3-min ‘all-out’ cycling test compared to a placebo [10]. Of particular interest is the lack of a difference in blood responses during experimental trials between the NaHCO_3_ doses in the present study. The absolute HCO_3_^−^ change from baseline was similar for both SBC conditions, as only a 0.5 mmol l^−1^ greater increase was observed in SBC3 compared to SBC2. Likewise, the increases in pH following NaHCO_3_ ingestion were similar between the SBC conditions. This may explain why no dose-dependent performance effects were observed in this study, as buffering capacity would have been increased to a similar extent and therefore lead to equal amounts of H^+^ buffering by circulating HCO_3_^−^. Moreover, in respect of blood lactate, a low TE range of 0.7 to 0.8% was displayed along with excellent ICC values (*r =* 0.91 to 0.92). This is in contrast to previous research displaying a TE of ~ 7% [6]. Nonetheless, some small inconsistencies were apparent as participants 6, 8 and 10 reported values with at least 2 mmol l^−1^ difference in SBC2, whilst participants 3 and 9 displayed this effect in SBC3. The reasons for this are unclear, although it may be due to the technical error associated with the lactate analyser used in this study [37]. Nonetheless, the present study displays that the physiological responses are consistent with repeated use of NaHCO_3_ meaning the primary acting mechanisms for performance enhancement should be in place if the athlete is seeking a performance enhancement.

A unique finding of the present study is that the performance responses following NaHCO_3_ were not dose dependent, akin to previous research [25]. The fastest 4-km TT completion time was observed in SBC2b although the other SBC treatments were not significantly slower (range + 0.2 to 0.9 s; % diff < 0.5%), suggesting a smaller dose of NaHCO_3_ may be plausible to prompt similar physiological and performance responses. A consideration of this study however is that no placebo treatment was utilised to identify if these responses were ergogenic. The central aim of the study was to quantify the reproducibility of performance following repeated NaHCO_3_ ingestion; therefore, this comparison was not included. Nonetheless, a control trial was conducted and our SBC conditions were significantly faster by 2.1 and 2.3% for SBC2 and 2.2 and 2.3% faster for SBC3. This suggests an ergogenic effect was apparent, in agreement with other studies using a time to peak alkalosis NaHCO_3_ ingestion strategy [10, 28]. Yet, further research should address this by comparing such performance responses to a placebo.

A practical benefit of utilising smaller doses of NaHCO_3_ is to minimise GI discomfort, as smaller doses have been shown to reduce the severity of symptoms [14, 26]. McNaughton [26] reported anecdotally as the amount of NaHCO_3_ ingested increased from 0.1 to 0.5 g kg^−1^ BM the severity of GI discomfort increased, however since this study, comparisons between doses have been sparse. Indeed, the severity and time to peak GI discomfort was reproducible in the present study using ICC analysis (ICC range *r* = 0.72 to 0.99); however, in some cases, the symptom suffered varied despite a similar rating of severity and time to peak. Participant 8 for instance suffered from nausea and bloating in SBC3a but then suffered diarrhoea and bowel urgency in SBC3b. The reasons for the discrepancies in symptoms remain inconclusive, as previous research seemed to suggest it was not linked to pre-exercise nutrition (carbohydrate, protein, fat and sodium intake) or the change in sodium (Na^+^) following NaHCO_3_ [14, 22], although further research is warranted. Nonetheless, this provides a challenge to the individual considering routine use of NaHCO_3_ as the symptom suffered may change with no clear pattern, which subsequently could impact the ability or desire to perform exercise, although the time to peak GI discomfort does seem to occur at the same time point. This influence of GI discomfort should therefore be monitored during training prior to use in competition.

## Conclusions

This study is the first to display consistent performance responses from NaHCO_3_ ingestion when exercise begins at the individual time of peak HCO_3_^−^. These datum support the use of individualised NaHCO_3_ supplementation strategies prior to performance to elicit reproducible physiological and performance responses. The use of personalised nutrition to maximise the bioavailability of HCO_3_^−^ and produce reliable responses in exercise that is competitive in nature is therefore recommended. Accompanying this finding, both 0.2 and 0.3 g kg^−1^ BM NaHCO_3_ produced similar blood acid-base balance and performance reliability, with no difference between doses, suggesting both amounts can be used as an ergogenic strategy. Lastly, the use of NaHCO_3_, irrespective of dose and repeated ingestion, appears to have a varied response on GI discomfort. Athletes should therefore monitor these responses, in an attempt to mitigate the impact of GI discomfort on exercise performance.
